# Cobertura y fidelidad de la prueba Xpert MTB/RIF™ en un área de alta carga de tuberculosis pulmonar en Colombia

**DOI:** 10.7705/biomedica.5272

**Published:** 2020-12-10

**Authors:** Freddy Agredo, Lyda Osorio

**Affiliations:** 1 Secretaría de Salud Municipal de Santiago de Cali, Cali, Colombia Secretaría de Salud Municipal de Santiago de Cali Cali Colombia; 2 Doctorado en Salud, Universidad del Valle, Cali, Colombia Universidad del Valle Universidad del Valle Cali Colombia; 3 Grupo de Epidemiología y Salud Poblacional, Escuela de Salud Pública, Universidad del Valle, Cali, Colombia Universidad del Valle Universidad del Valle Cali Colombia

**Keywords:** tuberculosis pulmonar, técnicas de diagnóstico molecular, rifampicina, resistencia a medicamentos, Tuberculosis, pulmonary, molecular diagnostic techniques, rifampin, drug resistance

## Abstract

**Introducción.:**

La prueba Xpert MTB/RIF™ es una prueba molecular rápida para el diagnóstico de la tuberculosis y la resistencia a la rifampicina. Desde el 2010 es la recomendada por la Organización Mundial de la Salud (OMS) y, aunque fue introducida en Colombia en el 2012, se desconocen los resultados de su uso.

**Objetivo.:**

Describir la cobertura y la fidelidad en el uso de la prueba Xpert MTB/RIF™ en pacientes con tuberculosis pulmonar en una ciudad con alta carga de la enfermedad en Colombia.

**Materiales y métodos.:**

Se hizo un estudio retrospectivo descriptivo de casos del programa de tuberculosis en Cali entre el 2013 y el 2019. La cobertura se estimó como el total de pruebas empleadas en los casos registrados en el programa. La fidelidad se midió con base en los protocolos internacionales de uso de la Xpert MTB/RIF™. Además, se hizo un análisis de correspondencias múltiples entre la prueba y las variables sociodemográficas.

**Resultados.:**

Se incluyeron 6.328 pacientes con tuberculosis pulmonar, de los cuales 181 eran resistentes a los fármacos. La cobertura total de la Xpert MTB/RIF™ durante el periodo de estudio fue de 10,3 % (n=655), con una variación anual entre 0,2 y 23 %. La fidelidad fue de 46,8 % para los grupos de mayor riesgo de tuberculosis multirresistente (TB-MDR). El uso de la prueba se relacionó con la condición de ser hombre, afrocolombiano, y tener entre 41 y 60 años de edad.

**Conclusiones.:**

La cobertura de la prueba Xpert MTB/RIF™ en Cali es baja y su uso no responde a la priorización recomendada para su implementación. Se requieren estrategias para promover su uso adecuado, de manera que contribuya a la meta de poner fin a la tuberculosis.

## Abstract (en)

**Introduction:**

The Xpert MTB/RIF™ is a rapid molecular test that diagnoses tuberculosis and rifampin resistance. Since 2010, it is recommended by the World Health Organization (WHO) and although it was introduced in Colombia since 2012, the results of its implementation are unknown.

**Objective:**

To describe the coverage and fidelity in the implementation of the Xpert MTB/ RIF™ in patients with pulmonary tuberculosis in a city with a high burden for the disease in Colombia.

**Materials and methods:**

We conducted a retrospective, descriptive study of cases from a tuberculosis program in Cali between 2013 and 2019. We estimated the coverage as the total number of tests used compared to the cases registered in the program and the fidelity based on international Xpert MTB/RIF™ implementation protocols. We performed a multivariate analysis of multiple correspondences between the test and the sociodemographic variables.

**Results:**

We included 6,328 patients with pulmonary tuberculosis of whom 181 were drug-resistant. The Xpert MTB/RIF™ coverage was 10,3% (n=655) with an annual variation between 0.2% and 23%. Loyalty among the highest risk groups of MDR-TB was 46.8%. The use of the test was related to being an Afro-Colombian man between 41 and 60 years of age.

**Conclusions:**

The coverage of the Xpert MTB/RIF in Cali is low and its use does not follow the recommended prioritization for its implementation. Implementation strategies are required for its proper use to contribute to the goal of ending tuberculosis.

La tuberculosis es una de las diez principales causas de muerte a nivel mundial y la principal causa de mortalidad en personas positivas para el HIV ^1^. La estrategia 'Alto a la TB" de la Organización Mundial de la Salud (OMS), adoptada por la Asamblea Mundial de la Salud en mayo del 2014, ofrece a los países un modelo para poner fin a la epidemia de la enfermedad reduciendo su mortalidad e incidencia y eliminando los costos catastróficos que conlleva ^2^.

La meta de poner fin a la epidemia en el 2030 es parte de los Objetivos de Desarrollo Sostenible en el área de la salud adoptados en el 2015. La OMS dio otro paso más y estableció la meta de reducir las tasas de mortalidad e incidencia en el 95 y el 90 % para el 2035, respectivamente, y situarlas en niveles similares a los actuales en los países con baja incidencia de la enfermedad ^3^. Se ha estimado que en el 2018 10 millones de personas enfermaron de tuberculosis y 1,5 millones murieron a causa de la enfermedad; más del 95 % de estas muertes se produjeron en países de ingresos bajos y medios ^4^.

Según la Organización Panamericana de la Salud (OPS), en el 2017 se notificaron 228.943 casos entre nuevos y recaídas de la enfermedad en el continente americano, lo que corresponde al 82 % de los casos estimados. La heterogeneidad de Latinoamérica y el Caribe dificulta la adaptación e implementación de las acciones para la eliminación y en ellos persiste la brecha entre los casos estimados y los notificados (no detectados o no notificados). Además, la poca accesibilidad al diagnóstico bacteriológico y especialmente al molecular, así como a las pruebas de sensibilidad a los fármacos que la combaten, sigue siendo un obstáculo para alcanzar las metas en ciertos países y territorios, lo que dificulta el diagnóstico oportuno y el tratamiento apropiado de la tuberculosis como tal y, específicamente, de la multirresistente (TB-MDR) y la extremadamente resistente (TB-XDR), así como la asociada con el HIV y la diabetes mellitus. Todo ello, aunado a la brecha en el diagnóstico de los casos, llevó a su aumento durante el 2016 y el 2017 ^5^.

En el 2018, en Colombia, se notificaron 14.338 casos de tuberculosis sensible (tasa de 26,0 por 100.000 habitantes) y 409 casos de tuberculosis resistente (tasa de 0,8 por casa 100.000 habitantes), de los cuales 11.647 eran nuevos ^6^. En Cali anualmente se presenta un promedio de 1.000 casos y muere una persona cada 4,5 días, con un promedio de pacientes multirresistentes de 13 por año ^7^.

La tuberculosis multirresistente constituye una crisis de salud pública y una amenaza para la seguridad sanitaria ^2^^,^^4^^,^^8^. En el 2018, la OMS estimó 484.000 casos nuevos de tuberculosis resistente a la rifampicina (TB-RR), de los cuales el 78 % era multirresistente ^4^. En el 2017 se registraron en las Américas 11.000 casos resistentes a la rifampicina (TB-RR) o a la rifampicina y la isoniazida (TB-MDR), de los cuales solo el 37 % fue notificado, lo que deja 6.900 casos de tuberculosis resistente y multirresistente (TB-RR/MDR) no diagnosticados ni tratados. Cinco países de las Américas concentran el 70 % de los casos estimados de TB-RR y MDR (Perú, Brasil, México, Ecuador y Haití). Por su parte, Colombia registró 570 casos, es decir, el 4 % del total de casos de la enfermedad en el país y 76,7 % acumulado frente al total de casos de las Américas ^5^. En diversos estudios sobre resistencia a medicamentos antituberculosos en Colombia entre 1995 y el 2007 se evidenció una tasa de resistencia inicial a los medicamentos de primera línea de 2,4 % ^9^.

En un estudio realizado en el 2004 con pacientes del Valle del Cauca, Moreira, *et al.,* reportaron una prevalencia de 6 % de tuberculosis resistente y una tendencia de resistencia primaria a los medicamentos de primera línea, principalmente en el municipio de Buenaventura ^10^, lo que queda corroborado con la elevada prevalencia de la resistencia a los antibióticos de primera línea como la isoniacida (94,2 %), el etambutol (25 %), la rifampicina (78,8 %) y la pirazinamida (21,2 %).

Según la OMS, en la región de las Américas hay una población de "alto riesgo" ^9^ expuesta a la circulación de cepas multirresistentes a fármacos de primera línea, siendo de 36,5 % los casos de resistencia a la isoniacida y a la rifampicina ^11^. Sin embargo, se estima que apenas el 54 % de los enfermos con TB-MDR recibe actualmente un tratamiento eficaz ^8^, siendo el acceso universal a las pruebas de sensibilidad a las drogas un aspecto clave en la meta de mejorar la detección de casos de TB-RR y MDR; por ejemplo, en el 2017 solo el 33 % de los pacientes recibió dichas pruebas ^5^.

Aunque recientemente la guía de tratamiento de la TB-MDR de la OMS avaló el tratamiento acortado (9 a 12 meses), su aplicación se ve limitada por la baja cobertura de las pruebas de detección de la resistencia a los antimicrobianos ^2^. Asimismo, el diagnóstico de los casos se hace por confirmación de laboratorio (73,9 % en el 2015). El tiempo entre el inicio de los síntomas y del tratamiento es variable. En un estudio del 2016 en ocho ciudades colombianas se encontró que dicho periodo fue de 51 días en promedio (27 a 101 días), con tiempos mayores a 30 días en el 72 % de los casos. El éxito del tratamiento (casos curados y tratamientos terminados) en los casos nuevos y en recaídas en el 2014 fue de 71 % y entre los casos con baciloscopia positiva, de 78,2 %, distante de la meta de 85 % propuesta por la OMS ^12^.

Históricamente, la dificultad del diagnóstico ha sido un obstáculo para dar una respuesta eficaz al control de la tuberculosis, a la infección simultánea con el HIV y a la TB-MDR ^13^. La baciloscopia es el método tradicional por su relativa facilidad, bajo costo y accesibilidad; sin embargo, es poco sensible (40-60 %) y no detecta la TB-MDR ^14^. El cultivo en medio sólido aumenta la sensibilidad y especificidad de la baciloscopia, pero tiene un mayor costo y los resultados demoran de seis a ocho semanas ^13^. El cultivo en medio líquido es más rápido y los resultados se demoran entre 11 y 14 días ^15^.

La prueba Xpert MTB/RIF™ recurre a la reacción en cadena de la polimerasa (PCR) en tiempo real y fue recomendada por la OMS desde el año 2010. Es capaz de detectar simultáneamente la presencia de la tuberculosis y la resistencia a la rifampicina (TB-RR) en un plazo de dos horas ^16^. En adultos, su sensibilidad se ha estimado en 98 % en individuos positivos por baciloscopia y cultivo, en 89 % como prueba inicial, en 67 % de los negativos por baciloscopia, y en 79 % de los positivos para HIV, con especificidades superiores al 98 % ^17^. Aunque es menor que en los adultos, la sensibilidad de la prueba en niños es superior a la de la baciloscopia para el diagnóstico de tuberculosis pulmonar ^18^. El despliegue mundial de la Xpert MTB/RIF™ ha cambiado el panorama del diagnóstico de la tuberculosis ^19^.

En Colombia, esta prueba se introdujo a finales del 2012 y en Cali en el 2013 como iniciativa de instituciones de salud del sector privado, y alcanzó a contar con cuatro equipos en el 2017. Con el fin de difundir algunos de los resultados de la implementación de esta prueba en esta ciudad, en el presente estudio se describen la cobertura y la fidelidad en el uso de la prueba Xpert MTB/RIF™ en pacientes con diagnóstico de tuberculosis pulmonar entre el 2013 y el 2017.

En este sentido, cabe señalar que la investigación clínica y la "investigación de la implementación" *(implementation research)* son diferentes ^20^, y que por los desafíos que enfrenta la comunidad mundial de la salud para pasar de las evidencias a la práctica, es imperativo incorporar nuevos marcos conceptuales y metodológicos. En ese sentido, la investigación de la implementación es el proceso de indagación científica de cuestiones relativas a la implementación de intervenciones, políticas, programas, prácticas, servicios e iniciativas de eficacia demostrada, orientadas a mejorar la salud de las poblaciones ^20^^-^^22^.

Se definió la cobertura como el número de personas con acceso a la prueba diagnóstica Xpert MTB/RIF™, es decir, el alcance que tiene la intervención, en tanto que la fidelidad se planteó como el grado de cumplimiento de la formulación inicial de la intervención en su ejecución, lo que refleja la calidad de la ejecución del programa y su capacidad de cumplir con el objetivo de desplegar en la práctica las acciones que tienen respaldo en la evidencia ^20^^-^^22^.

## Materiales y métodos

### Diseño y población de estudio

Se hizo un estudio descriptivo retrospectivo de los registros de las bases de datos del programa de tuberculosis de la Secretaría de Salud de Cali y del sistema de vigilancia (Sivigila) entre el 2013 y 2019 ^23^. Se incluyeron todos los registros de hombres y mujeres de cualquier edad con diagnóstico de tuberculosis pulmonar residentes en el municipio de Cali y se excluyeron los casos extrapulmonares y los remitidos de otras ciudades.

### Procedimientos

A partir de los registros del programa se seleccionaron las variables correspondientes a la información del paciente, el diagnóstico y el seguimiento bacteriológico. La información se completó y se verificó comparándola con la base de datos del Sivigila.

La calidad de la información se validó con los datos del libro de sintomáticos respiratorios, el libro de registro diario de baciloscopia y cultivo, la tarjeta individual de tratamiento, el informe de casos y actividades, el informe de cohortes y el consolidado trimestral de actividades de bacteriología disponibles en el programa de tuberculosis. Estos libros y la tarjeta son de diligenciamiento obligatorio para las instituciones de salud que atienden a los pacientes con tuberculosis no confirmada y deben ser reportados al ente territorial, el cual es responsable de consolidar la información del municipio de Cali.

### Análisis estadístico

Se hizo el análisis descriptivo de la frecuencia de casos por año, los grupos de edad, el sexo, el régimen de afiliación al sistema de seguridad social, la condición de vulnerabilidad (farmacodependencia y drogodependencia, privación de la libertad y habitante de calle), las comorbilidades (infección simultánea con el virus del HIV, diabetes mellitus y estado nutricional), los resultados de las pruebas diagnósticas de ingreso y seguimiento y de las de sensibilidad a los antimicrobianos.

Se definió la cobertura de la Xpert MTB/RIF™ como el número de personas a quienes se les hizo la prueba comparado con el número de pacientes inscritos en el programa durante el mismo periodo de tiempo.

La fidelidad se definió como el grado en que la Xpert MTB/RIF™ se implemento según el protocolo de la OMS del 2014. Con base en estos criterios y la realidad propia del país se consideraron los siguientes cuatro grupos.

1. Grupo A: individuos sospechosos de tener tuberculosis con baciloscopia negativa y cultivo positivo. En este grupo se incluyeron las personas cuya condición en el momento del ingreso se caracterizaba por abandono, fracaso, recaída (si el resultado de su baciloscopia era positivo a los dos meses) o paciente nuevo (si la baciloscopia era negativa a los dos meses, pero daba positivo a los cuatro, seis o 12 meses.
2. Grupo B: personas sospechosas de tener tuberculosis asociada con el HIV, es decir, pacientes positivos para el virus, privados de la libertad o habitantes de calle.
3. Grupo C: personas sospechosas de tener tuberculosis con antecedentes epidemiológicos de alta probabilidad de resistencia a la rifampicina, entre quienes se incluían personas extranjeras y migrantes venezolanos. En el presente estudio se decidió considerar dentro de este grupo a los migrantes, los consumidores de sustancias psicoactivas y los habitantes de calle.

**Figura 1:**
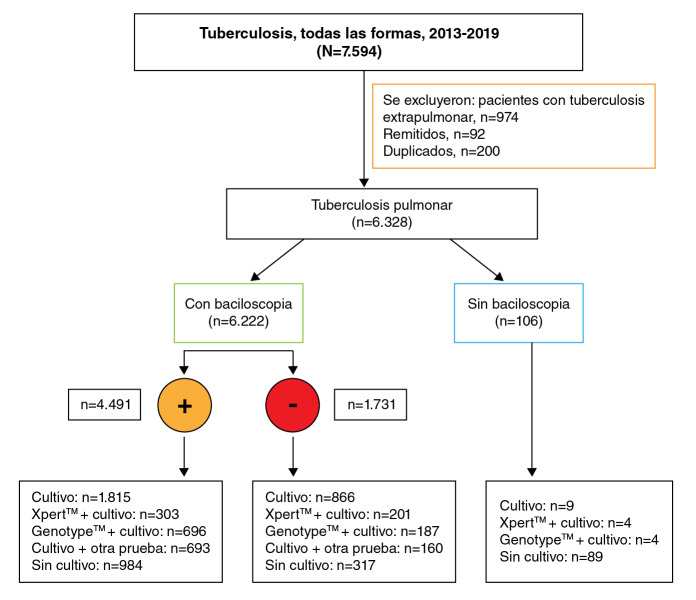
Flujograma de selección y diagnóstico microbiológico de pacientes con tuberculosis pulmonar, Cali, 2013-2019

Si bien la OMS clasifica un cuarto grupo, el D, también llamado grupo especial, que incluye a todas las personas sospechosas de tener tuberculosis (adultos y niños), en las que la prueba Xpert MTB/RIF™ puede usarse como una prueba de diagnóstico inicial de la enfermedad, y a pacientes con sospecha de meningitis tuberculosa, que en este estudio se excluyeron por tratarse de pacientes con tuberculosis extrapulmonar (figura 1) ^24^.

Las diferencias en la frecuencia de las variables categóricas entre los grupos se determinaron con la prueba de ji al cuadrado. Los valores de p menores de 0,05 se consideraron estadísticamente significativos. La relación entre las variables de uso de la Xpert MTB/RIF™, régimen de afiliación (forma de afiliación al Sistema General de Seguridad Social en Salud en Colombia: el subsidiado que cubre a la población pobre sin capacidad de pago que accede a los servicios de salud mediante un subsidio estatal; el contributivo, que cubre a las personas con capacidad de pago y con vinculación laboral, y el de excepción o especial, que cubre al personal de las fuerzas militares y de policía y de la justicia penal militar, así como a los profesores pertenecientes al magisterio, los servidores públicos de Ecopetrol y sus beneficiarios) ^25^.

Además del análisis descriptivo, se hizo un análisis de correspondencias múltiples, método descriptivo o exploratorio cuyo objetivo es resumir una gran cantidad de datos en un número reducido de dimensiones con la menor pérdida de información posible, en lo cual se asemeja a los métodos factoriales, salvo que el primero se aplica a variables categóricas u ordinales e implica el tratamiento de la proximidad o distancia entre los elementos a partir de un punto en el espacio, de forma que las relaciones de cercanía o lejanía entre los puntos calculados reflejen las relaciones de dependencia y semejanza existentes entre las variables.

La existencia o no de algún tipo de relación entre las variables X y Y se analiza mediante contrastes de las hipótesis frente a su independencia. En este estudio se agruparon en dos dimensiones: en la primera se agruparon las variables que describían las condiciones de atención (régimen de afiliación y tipo de prueba) y en la segunda, las variables sociodemográficas. Los datos se manejaron en MS Excel 2007 antes de ser exportados a Epi Info, versión 7.2, y SPSS™, versión 22, para su análisis.

## Resultados

### Características de los pacientes

Durante el periodo de estudio se registraron 7.594 pacientes con diagnóstico de tuberculosis en todas sus formas de presentación; de ellos se excluyeron 200 repetidos, 92 remitidos y 974 con tuberculosis extrapulmonar, y se incluyeron 6.328 registros (83,3 %) de pacientes con tuberculosis pulmonar. Los 974 pacientes excluidos tenían tuberculosis extrapulmonar de tipo pleural (n=455), ganglionar (n=173), meníngea (n=114), peritoneal (n=44), miliar (n=20), intestinal (n=18), laríngea (n=2) y en otros sitios (n=148).

La baciloscopia se había hecho en el 98,3 % (6.222) de los pacientes con tuberculosis pulmonar: 4.491 (72,2 %) fueron positivos y 1.731 (27,8 %), negativos. Se hizo cultivo en 4.154 (65 %) de los 6.328 pacientes, de los cuales fueron positivos 1.875 (54,5 %), negativos 1.858 (40,2 %), en tanto que las muestras estaban contaminadas en 27 (0,7 %) y no hubo reporte en 289 (4,6 %) (figura 1).

En los siete años de estudio (2013-2019) ingresaron en promedio 904 casos por año, con una tendencia creciente, dándose el mayor número de registros en el 2017 (1.017 casos, 23,3 %). El total de casos nuevos fue de 5.303 (83,8 %) y las recaídas fueron 362 (5,7 %).

La mayoría de los pacientes correspondía al sexo masculino (63,8 %), tenía entre 25 y 34 años de edad (26,7 %), 3.020 (47,7%) pertenecían al régimen subsidiado, 583 (9,2 %) pacientes tenían HIV, en 585 (9,29 %) casos había desnutrición, 814 (12,9 %) estaban privados de la libertad, 550 (8,7 %) eran consumidores de sustancias psicoactivas y 339 (5,4 %) eran habitantes de la calle.

En cuanto a los casos de tuberculosis resistente, entre el 2013 y el 2019 se detectaron 181, 13 (7,2 %) de ellos en el 2013, 26 (14,4 %) en el 2014 y otros tantos en el 2015, 32 (35,4 %) en el 2016 y los mismos en el 2017, 35 (19,3 %) en el 2018, año con la mayor detección de casos resistentes, y 17 (9,4 %) en el 2019. La mayoría de estos pacientes eran hombres (n=120, 66,7 %) (cuadro 1). Asimismo, se encontró que de los 181 casos, 128 (70,7 %) eran monorresistentes, 52 (28,7 %) tenían tuberculosis multirresistente y uno (0,6 %) era polirresistente.

**Cuadro 1:** Características de pacientes con tuberculosis pulmonar y tuberculosis resistente, Cali, 2013-2019

Características	Tuberculosis pulmonar (n=6.328) n (%)	Tuberculosis resistente Farmacoresistente FfFarmacorresist (n=181)
Año de registro		
2013	795 (12,6)	13 (7,2)
2014	818 (12,9)	26 (14,4)
2015	808 (12,8)	26 (14,4)
2016	919 (14,5)	32 (17,7)
2017	1.017 (16,1)	32 (17,7)
2018	1.012 (16)	35 (19,3)
2019	959 (15,2)	17 (9,4)
Condición de ingreso		
Nuevo	5.303 (83,8)	139 (76,8)
Recaída	362 (5,7)	23 (12,7)
Fracaso	327 (5,6)	11 (6,1)
Abandono	132 (2,1)	7 (3,9)
Remitido de otra ciudad	174 (2,7)	1 (0,6)
Sexo		
Femenino	2.168 (36,2)	61 (33,3)
Masculino	4.160 (63,8)	120 (66,7)
Edad (años		
0-14	220 (3,5)	1 (0,6)
15-19	266 (4,2)	6 (3,3)
20-24	866 (13,7)	28 (15,5)
25-29	823 (13)	16 (8,8)
30-34	655 (10,4)	25 (13,8)
35-39	488 (7,7)	23 (12,7)
40-44	385 (6,1)	11 (6,1)
45-49	406 (6,4)	7 (3,9)
50-54	475 (7,5)	14 (7,7)
55-59	433 (6,8)	12 (6,6)
60-64	399 (6,3)	10 (5,5)
Más de 64	912 (14,4)	28 (15,5)
Régimen^+^		
Contributivo	2.083 (32,9)	54 (30)
Subsidiado	3.020 (47,7)	96 (53)
No asegurado	639 (10,1)	21 (11,6)
Especial	503 (7,9)	8 (4,4)
Excepción	83 (1,3)	2 (1,1)
Condición de vulnerabilidad		
Consumidor de sustancias psicoactivas	550 (8,7)	18 (9,9)
Privado de la libertad	814 (12,9)	15 (8,3)
Otra vulnerabilidad	1.013 (16)	23 (12,7)
Habitante de calle	339 (5,4)	11 (6,1)
Ninguna	3.612 (57,1)	114 (63)
Comorbilidad		
Diabetes	487 (7,7)	19 (11,6)
HIV	583 (9,2)	21 (13,2)
Desnutrición	585 (9,2)	21 (15,5)

Los tipos de resistencia a los medicamentos antituberculosos son, monorresistencia: resistencia a solo un antituberculoso de primera línea; polirresistencia: resistencia a más de un antituberculoso de primera línea distinto de la isoniazida y la rifampicina; multirresistencia: resistencia por lo menos a la isoniazida y la rifampicina.

^+^ Formas de afiliación al Sistema General de Seguridad Social en Colombia (régimen subsidiado, contributivo y de excepción o especial y no asegurado)

### Cobertura y fidelidad de la prueba Xpert MTB/RIF™

**Figura 2:**
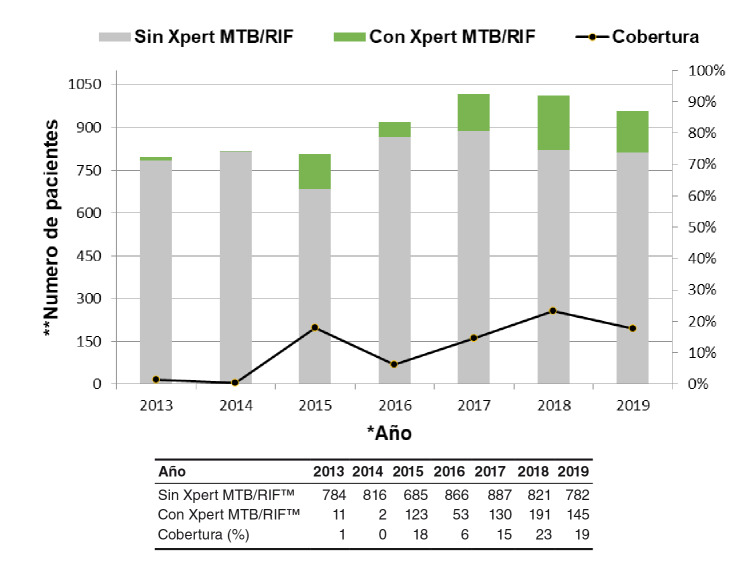
Cobertura de Xpert MTB/RIF™ en pacientes con tuberculosis pulmonar, Cali, 2013-2019

^*^ Eje de las X: años ^**^ Eje de las Y: número de pacientes ^***^ Cobertura: expresada en porcentaje

De los 6.328 casos de pacientes con tuberculosis pulmonar detectados entre el 2013 y el 2019, la prueba Xpert MTB/RIF™ se le hizo a 655 pacientes, es decir, hubo una cobertura promedio de 10,35 %, la cual varió anualmente: en el 2013 se le práctico a 11 (1 %) de 784 pacientes; en el 2014 no se le hizo la prueba a ninguno de los 816 pacientes porque las muestras se enviaron al Hospital Universitario de la ciudad para la prueba Genotype MTBDRplus™; en el 2015 se le hizo a 123 (18 %) de los 685 pacientes; en el 2016 a 56 (6 %) pacientes de 866; en el 2017 a 130 (15 %) de 887 pacientes; en el 2018, año de la mayor cobertura, a 191 (23 %) de 821 pacientes, y en el 2019 a 145 (19 %) de 782 pacientes (figura 2).

Otras pruebas como la Genotype™ y el cultivo en medio líquido MGIT tuvieron una cobertura de 1.156 casos (18,36 %) y 480 (7,6 %), respectivamente. Sin embargo, la realización de las pruebas se asoció con el régimen de afiliación a salud (p<0,001) y al sexo (p<0,001) en la prueba de ji al cuadrado, observándose una mayor frecuencia de uso de la Xpert MTB/ RIF™ en el régimen contributivo (63,4 %) y en mujeres (42 %), en tanto que la Genotype™ y el cultivo en MGIT fueron más frecuentes en el régimen subsidiado (56,6 y 62,7 %, respectivamente) y en hombres (66,3 y 68,1 %, respectivamente) (cuadro 2).

**Cuadro 2:** Relación del uso de pruebas de sensibilidad con el régimen de salud y el sexo en pacientes con tuberculosis pulmonar, Cali, 2013-2019

Característica	Xpert MTB/RIF™ (n=655) n (%)	Genotype™ (n=1.156) n (%)	Cultivo en MGIT (n=480) n (%)	Otra prueba^*^ (n=303) n (%)	pχ^2^
Régimen^+^					
Subsidiado	197 (30)	654 (56,5)	301 (63)	127 (42)	<0,001
Contributivo	415 (63)	303 (26)	64 (13)	26 (8,5)	
Especial	7 (1)	39 (4,0)	42 (9)	132 (43)	
No asegurado	29 (6)	157 (13,5)	73 (15)	11 (6,5)	
Sexo^++^					
Masculino	380 (58)	767 (66)	327 (68)	259 (85)	<0,001
Femenino	275 (42)	389 (34)	153 (32)	44 (15)	

^*^ Otra prueba: se refiere a criterio epidemiológico, rayos X de tórax, IGRA

^+^ Régimen de salud, valor de p<0,001; prueba de ji al cuadrado de Pearson=1038

Razón de verosimilitud=840; asociación lineal=5,873; gradiente de libertad=16

^++^ Sexo, valor de p<0,001; prueba de ji al cuadrado de Pearson=72

Razón de verosimilitud=79,6; asociación lineal=1,245; gradiente de libertad=4

**Figura 3:**
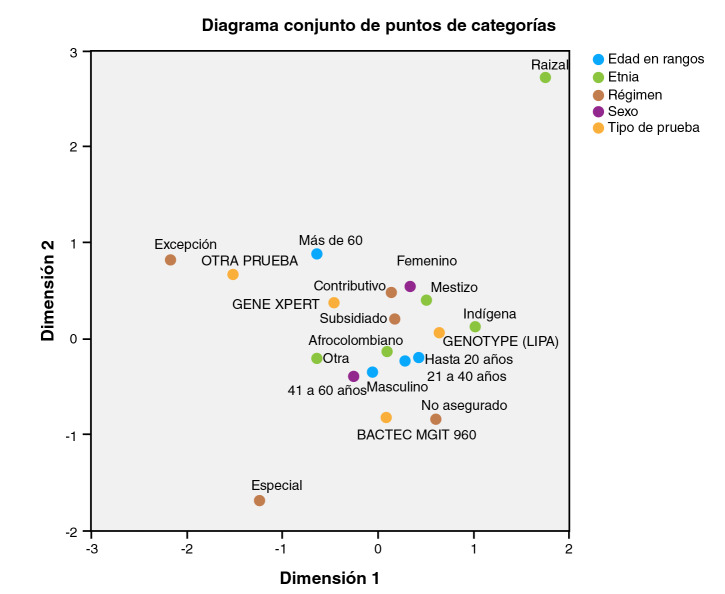
Análisis de correspondencias múltiples de variables de atención y sociodemográficas en pacientes con tuberculosis pulmonar, Cali, 2013-2017

^*^ Dimensión 1: variables que describen condiciones de atención (régimen de afiliación y tipo de prueba) ^**^ Dimensión 2: variables sociodemográficas ^***^ La prueba de hipótesis utilizada fue la de ji al cuadrado de Pearson. (Ho: ambas variables son independientes; Ha: existe una relación de dependencia

En el análisis de correspondencias múltiples se evidenció una relación similar entre el régimen contributivo y el subsidiado en cuanto al uso de la Xpert MTB/RIF™, pero más estrecha con el ser afrocolombiano, hombre y tener entre 41 y 60 años de edad. Las asociaciones más fuertes se encontraron entre el régimen de excepción y otros tipos de pruebas diferentes a la Xpert MTB/RIF™, entre la Genotype™ y el cultivo en MGIT y el ser mayor de 60 años, entre la Genotype™ y la población indígena y ser menor de 40 años (figura 3).

En cuanto a la fidelidad en la implementación de la Xpert MTB/RIF™, se observaron frecuencias de uso casi similares en los grupos A y B y, en menor proporción, en el grupo C. En los últimos siete años, en el grupo B se registró un mayor uso, excepto en el 2013 (cuadro 3).

**Cuadro 3:** Frecuencia de Xpert MTB/RIF™ por grupo de implementación propuesto por la Organización Mundial de la Salud en pacientes con tuberculosis pulmonar, Cali, 2013-2019

Año	2013	2014	2015	2016	2017	2018	2019	Total (n=6.328) n (%)
A^*^	174	183	183	168	194	183	141	1.220 (19,4)
B^**^	143	187	204	219	296	339	261	1.649 (26,1)
C^***^	15	26	26	32	32	37	17	181 (2,9)
D^****^	5	13	29	22	23	20	10	122 (1,9)

^+^ Grupos según la clasificación de la OMS, 2014

* Pacientes con sospecha de tener tuberculosis con baciloscopia negativa y cultivo positivo

^**^ Pacientes con sospecha de tener tuberculosis asociada con HIV

^***^ Pacientes con sospecha de tuberculosis y antecedentes de alta probabilidad de resistencia a la rifampicina

^****^ Pacientes con diagnóstico de tuberculosis meníngea. Este grupo fue excluido del análisis porque en este estudio solo se tuvieron en cuenta a los pacientes con tuberculosis pulmonar.

## Discusión

En los últimos años varios estudios han evaluado la Xpert MTB/RIF™, particularmente su desempeño diagnóstico y costo-efectividad en los casos de tuberculosis y tuberculosis multirresistente, y, en menor medida, los resultados y el impacto de su implementación ^26^^-^^31^. El presente estudio evaluó dos resultados de implementación, la cobertura y la fidelidad, desde su introducción en Cali en el 2013. Los resultados evidenciaron una baja cobertura y fidelidad teniendo como referentes los grupos priorizados por la OMS en su manual de implementación del 2014 ^24^.

En una evaluación de la implementación de la Xpert MTB/RIF™ entre el 2012 y el 2014 en 18 centros clínicos de Uganda se demostró que el volumen de pruebas fue bajo: solo el 8 % de la capacidad de utilización de la prueba y el 21 % de las personas con síntomas de tuberculosis, y únicamente en una muestra de esputo negativa se hizo la prueba. En ese estudio, el desabastecimiento de cartuchos, el mal funcionamiento del módulo y las tasas de error consistentemente altas fueron los problemas más comunes ^29^. Se desconoce si estos factores afectan la baja cobertura de la prueba en Cali.

En Mozambique y Suazilandia, la Xpert MTB/RIF™ se utilizó en menos de dos tercios de la capacidad instalada, a pesar de tener una mayor carga de tuberculosis y haber desplegado una red de transporte de muestras, haber coordinado las redes de suministro, haber capacitado al personal de laboratorio y supervisado el proceso ^30^^,^^31^. Estos hallazgos reflejan especialmente la implementación real en la mayoría de los entornos de bajos ingresos, lo que subraya el hecho de que el uso de una prueba novedosa no necesariamente mejora los resultados del paciente. Además de la infraestructura de soporte, los programas deben considerar la posibilidad de probar algoritmos que aprovechen la capacidad existente y el volumen de pruebas previstas, así como de evaluar diferentes estrategias de implementación ^29^.

La cobertura de la Xpert MTB/RIF™ ha cambiado en los últimos cinco años en Cali, en parte por el efecto que han tenido las intervenciones financiadas con fondos adicionales a los del programa, como los del Fondo Financiero de Proyectos de Desarrollo (FONADE) y el Fondo Mundial ^32^. Por otra parte, la cobertura de la prueba molecular Genotype™ es mayor que la de la Xpert MTB/RIF™ por ser la primera la que está disponible en el principal hospital de tercer nivel de la ciudad. Se desconocen las razones por las cuales esa institución adoptó la Genotype™ y no la Xpert MTB/RIF™, a pesar de que existe evidencia local de que esta tiene un mejor desempeño que la baciloscopia de esputo en casos sospechosos de tuberculosis en la comunidad y podría aumentar la confirmación de la tuberculosis pulmonar con un beneficio adicional en términos de costo-efectividad (Rosso F, Pacheco R. Comparación de costo y efectividad del uso de Xpert MTB-RIF en el diagnóstico de TB en tres instituciones del Valle del Cauca. Cuarto Congreso de la Asociación Colombiana de Economía en Salud: estudios en TB. Cali; 2013. p. 22).

La baja cobertura también se podría explicar, en parte, por la falta de directrices del nivel nacional o regional para la implementación de las pruebas moleculares en los programas de tuberculosis; en Colombia, solo hay dos directrices emanadas del Ministerio de Salud y Protección Social (circulares externas 0007 de 2015 y 055 de 2016) sobre el uso de pruebas moleculares ^33^^,^^34^. Dichas circulares señalan que cuando esté disponible en la entidad territorial y se ajuste a la contratación de las entidades promotoras de salud (EPS) y de las empresas del sistema de salud prestadoras de servicios médicos ^25^ responsables de la atención de la personas con tuberculosis e infección simultánea con HIV, se deberán ofrecer las pruebas moleculares para el diagnóstico oportuno dada su mayor sensibilidad en este grupo de pacientes, sin detrimento de las pruebas diagnósticas convencionales. A pesar de esta directriz, la utilización de la Xpert MTB/RIF™ en el grupo B, el cual incluye a aquellos con HIV, fue relativamente baja.

El manual de implementación de la Xpert MTB/RIF™ de la OMS del 2014 es una guía adaptable a diferentes contextos, y varios países en Europa y Latinoamérica, como Brasil, Salvador, Perú ,Chile y México, ya la han implementado ^19^Sunnyvale, CA, USA.

Los resultados en Western Cape, Sudáfrica, demuestran que, si bien la implementación de la Xpert MTB/RIF™ permitió más confirmaciones de casos de tuberculosis que la microscopía de esputo, los niveles de pérdida en el seguimiento y la mortalidad no se redujeron en quienes la recibieron. Sin embargo, la prueba no se utilizó como un verdadero servicio en el punto de atención ^35^. Los resultados de la Xpert MTB/RIF™, que se entregan a los pacientes en el momento y en el lugar de la recolección de la muestra, pueden ser diferentes a los que se entregan en el tiempo usual ^36^. En cuanto a los resultados de la investigación de la implementación, se consideraron la cobertura y fidelidad porque permiten analizar si la intervención está logrando los resultados operativos previstos en la población objetivo ^37^.

La adaptación de este manual de implementación de la Xpert MTB/RIF™ en Colombia, y específicamente en Cali, requiere una evaluación previa de las estrategias de implementación a la luz de los recursos disponibles para la compra de equipos e insumos, así como las formas de pago de la prueba en el marco de contratación vigente entre las EPS y las empresas administradoras de planes de beneficios en salud (EAPB), es decir, las entidades promotoras de salud de los regímenes contributivo y subsidiado, así como las empresas solidarias de salud, las asociaciones mutuales en sus actividades de salud, las entidades promotoras de salud indígenas, las cajas de compensación familiar en sus actividades de salud, las entidades que administren planes adicionales de salud, las entidades obligadas a compensar, las entidades adaptadas de salud, las entidades pertenecientes al régimen de excepción en salud y las universidades en sus actividades de salud ^25^^,^^38^.

La cobertura es la proporción de la población que recibe efectivamente la intervención e involucra el acceso a los servicios. Por su parte, la fidelidad es el grado en que la implementación de una intervención se ajusta a su formulación inicial ^39^ y refleja la ejecución del programa y su capacidad para mantener el objetivo de desplegar en la práctica real las acciones que tienen respaldo en la evidencia. Estas dos características se integran para brindar un servicio de calidad a los pacientes. No hay estudios que evalúen a la vez cobertura y fidelidad de la implementación de la Xpert MTB/RIF™, pero en el futuro deberán evaluarse algunos de los desafíos establecidos para la implementación de la prueba en países de ingresos bajos y medios: la falta de orientación y capacitación estandarizada del personal médico y de bacteriólogos, el control de la calidad en el laboratorio clínico, los procesos de planificación y mantenimiento de la máquina de procesamiento de la Xpert MTB/RIF™ y la adquisición de los cartuchos, los problemas de suministro de energía y las dificultades para registrar la información de los resultados ^19^.

Las recomendaciones de la OPS para el diagnóstico de la enfermedad prefieren el acceso universal a la investigación rápida de la tuberculosis y la tuberculosis resistente a la isoniazida (MDR y RR; algoritmo 1), seguido de la investigación rápida de los dos tipos de la enfermedad en grupos priorizados hasta alcanzar la cobertura universal (algoritmo 2), algoritmos que son los de referencia de la OPS ^40^. A pesar de estas recomendaciones, los resultados demuestran que en Cali se están realizando varias pruebas diagnósticas simultáneamente en el mismo paciente sin seguir un protocolo o algoritmo estandarizado. La Xpert MTB/RIF™ en Cali se destina principalmente a pacientes con riesgo de tuberculosis multirresistente e infección concomitante de tuberculosis y HIV y muy poco en quienes dan negativo en la baciloscopia.

Los datos obtenidos reflejan que la aplicación de la prueba depende más del régimen de salud y el sexo de los pacientes que de la priorización por grupos de riesgo. Esto se evidenció por la mayor cobertura de la prueba Genotype™ en la población con tuberculosis pulmonar que acude al hospital de mayor nivel de complejidad, así como de otras pruebas en sujetos afiliados al régimen especial (fuerzas militares, policía nacional, Ecopetrol, magisterio y universidades públicas).

Las características de los pacientes con tuberculosis pulmonar y de quienes presentaban el tipo resistente coincidieron con las reportadas por el Instituto Nacional de Salud de Colombia: ser del sexo masculino y tener entre 25 y 34 años de edad ^6^. Llama la atención que el 78 % de los casos de tuberculosis resistente correspondió a casos nuevos, lo que sugiere que el nexo epidemiológico de estos pacientes podría ser otro paciente con resistencia, por lo que la magnitud de la tuberculosis resistente estaría subestimada. Los casos de recaídas, fracasos y abandono indican la necesidad de fortalecer el control y el seguimiento durante el primer tratamiento. Además, en los pacientes positivos para el HIV con baciloscopia positiva, el resultado negativo en la Xpert MTB/RIF™ confirma que se trata de otra micobacteria, lo que es útil para orientar el tratamiento ^41^. En los últimos cinco años se han aislado en Cali 101 cultivos de micobacterias no tuberculosas, siendo las más frecuentes las del complejo *Mycobacterium avium, M. gordonae, M. xenopi, M. kansasii y M. fortuitum.*

En conclusión, la implementación de técnicas moleculares como la Xpert MTB/RIF™ para el diagnóstico de la tuberculosis resistente y multirresistente es relativamente reciente en el país. La cobertura y la fidelidad en su implementación en Cali es baja y su uso no sigue necesariamente la priorización recomendada. Deben evaluarse las estrategias de implementación para contribuir a su uso adecuado y al cumplimiento de las metas nacionales e internacionales para poner fin a la tuberculosis. Este estudio proporciona información de base para proponer mejoras en la implementación de alternativas de diagnóstico con pruebas moleculares ya disponibles en el país que contribuyan al plan estratégico "Colombia libre de tuberculosis, 2015-2035" ^32^.
